# Antifungal Potential of Some Herb Decoctions and Essential Oils on *Candida* Species

**DOI:** 10.3390/healthcare10101820

**Published:** 2022-09-21

**Authors:** Alexandra Noites, Bruno Araújo, Jorge Machado, Eugénia Pinto

**Affiliations:** 1 Laboratory of Applied Physiology, ICBAS—Institute of Biomedical Sciences Abel Salazar, University of Porto, 4050-313 Porto, Portugal; 2 Laboratory of Microbiology and Biological Sciences Department, FFUP-Faculty of Pharmacy, University of Porto, 4050-313 Porto, Portugal; 3 CBSin—Center of BioSciences in Integrative Health, 4250-105 Porto, Portugal; 4 CIIMAR—Interdisciplinary Centre of Marine and Environmental Research, 4450-208 Matosinhos, Portugal

**Keywords:** antifungal activity, *Coptis chinensis*, *Magnolia officinalis*, *Scutellaria barbata*, *Azadirachta indica*, *Leptospermum scoparium*, *Candida* spp.

## Abstract

(1) Background: Candidiasis is a fungal infectious disease caused by opportunistic *Candida* species. The incidence of candidiasis has improved, due to prolonged antibiotic therapy and an increased number of immunocompromised patients. The purpose of this study was to evaluate if decoctions and essential oil (EO) of neem (*Azadirachta indica*, Meliaceae family), coptidis (*Coptis chinensis*, Ranunculaceae family), magnolia (*Magnolia officinalis*, Magnoliaceae *family*), scutellaria (*Scutellaria barbata*, Lamiaceae family), and the EO of manuka (*Leptospermum scoparium*, Myrtaceae family), have antifungal activity in vitro against some clinically prevalent species of *Candida*. (2) Methods: The antifungal activity was studied by the determination of the minimum inhibitory concentration (MIC) and minimum lethal concentration (MLC) against five *Candida* strains. The effect in dimorphic transition of *Candida albicans* was also evaluated for the two plants with higher antimicrobial behavior. (3) Results: *C. chinensis* decoction and EO and *L. scoparium* EO exhibited antifungal activity in *Candida* spp. In addition to the fact that both *C. chinensis* decoction and EO proved strong antifungal activity, *L. scoparium* EO also displayed a relevant inhibitory effect on the dimorphic transition. (4) Conclusions: The results provided support for the potential use of *C. chinensis* and *L. scoparium* in the treatment of infections by *Candida* spp.

## 1. Introduction

In recent years, fungal infections have become relevant causes of morbidity and mortality, especially in immunocompromised patients. The increased use of antifungals has led to a higher and more diverse number of resistances to this type of drugs. In the quest for alternative therapies, there has been a greater interest in natural products with antifungal activity [1,2]. In addition to their single use, scientific research has also focused on the combined potential of these natural products with the classic antifungal agents for more efficient results. In addition to the efficacy issues, it is also beneficial that a natural product, alone or combined, exhibits fewer undesirable side effects, thereby promoting correct therapeutics [3]. Fungal infections caused by *Candida* spp. encompass a broad spectrum of conditions including superficial and systemic opportunistic diseases. In healthy individuals, they are often responsible for local symptoms in the skin or in the infected mucosae. In immunocompromised individuals with degenerative and/or neoplastic pathologies or in individuals carrying medical devices, infections already disseminated at a systemic level and that may affect internal organs are frequently reported [4,5]. Aside from the virulence factors of the fungi, the infection is dependent of numerous aspects inherent to the patient itself, so that has also to be taken in account [6]. *Candida albicans* has been reported as the most common pathological agent in about 60.0% of cases [4]. The first step in the infection by *C. albicans* is the adhesion to host cells or implanted medical devices and this is followed by hyphal growth and biofilm formation. This yeast-to-hyphal transition has long been identified as a key factor in their fungal virulence; therefore, it is necessary to develop new antifungals with inhibitory action on adhesion and yeast–hyphal transition by *C. albicans* [7]. However, other strains have emerged regarding their prevalence and must be considered in the choice of the most appropriate therapy [4]. *Candida* species such as *Candida glabrata, Candida krusei, Candida parapsilosis*, and *Candida tropicalis* infections are increasingly prevalent. In the future, non-albicans *Candida* species would pose more clinical problems, especially in compromised population groups, which might justify a further study [8]. Azoles, such as fluconazole and itraconazole, are the most frequently prescribed antifungals in candidiasis therapy. They destroy the cellular structures of fungi by inhibiting the biosynthesis of membranous ergosterol. However, long-term, or repeated exposure to azoles in refractory infections can induce the emergence of resistant strains [9]. Some of the non-*albicans Candida* species are rapidly becoming resistant to first- and second-line antifungals, such as fluconazole and echinocandins [10]. *C. krusei* is intrinsically fluconazole-resistant, while the resistance of *C. glabrata* may be acquired [9]. *C. glabrata* has emerged as the second most common cause of blood stream and mucosal infection in many countries, being naturally more resistant to fluconazole than *C. albicans* and easily developing further fluconazole resistance in the case of the prolonged therapy of patients [11]. Therefore, novel fungal therapies for effective management of *Candida* infections are required. Many plant extracts and essential oils (EOs) have biological activity both in vitro and in vivo, which has justified research on traditional medicine focused on the characterization of their antimicrobial activity [12,13]. Decoction is the traditional form of preparation of herbal medicine in traditional Chinese medicine (TCM) and EO has a pharmacological potential already demonstrated in numerous studies, mainly due to the presence of phenolic and terpenoid compounds, which have antibacterial or antifungal properties [14,15,16,17]. *Coptis chinensis, Magnolia officinallis*, and *Scutellaria barbata* are plants used in TCM. The main classic indications of *C. chinensis* include diabetes, stomach fulness and distention, gastroesophageal reflux, bacterial diarrhea, high fever, jaundice, palpitations, hyperactivity, anxiety and insomnia, blood spitting or nose bleeding, red eyes, and toothache [18]. The wide range of applications of *M. officinalis* bark includes the treatment of gastrointestinal disorders, anxiety, depression, nervous disorders, asthma, and allergic disease, as well the relief of headaches, muscular pain, and fever [19]. TCM has used *S. barbata* to treat a spectrum of diseases including hepatitis, appendicitis, pain, pulmonary abscess, and ascites due to cirrhosis and cancer [20]. *Azadirachta indica* is used by traditional Ayurveda medicine for the treatment of acne, fever, leprosy, malaria, ophthalmia, and tuberculosis [21]. *Leptospermum scoparium* has been used historically in traditional Maori medicine for a variety of infection-related conditions including urinary tract conditions, intestinal complaints, coughs, colds, skin conditions such as burns, and is used as a mouthwash in gum disease [22]. As these five plant products have been used traditionally as antiseptics or antimicrobials, this study was designed to scientifically investigate their possible antifungal activity.

## 2. Materials and Methods

### 2.1. Standards and Reagents

Dimethyl sulfoxide (DMSO) and 3-(N-morpholino) propanesulfonic acid (MOPS) were purchased from Sigma-Aldrich (St. Louis, MO, USA). Sodium hydroxide (NaOH) was from Panreac (Barcelona, Spain). Pfizer (New York, NY, USA) kindly provided voriconazole. Sabouraud dextrose agar (SDA) and API ampoules were purchased from BioMérieux (Marcy L’Etoile, France). RPMI- 1640 broth medium (with L-glutamine, without bicarbonate, and with the pH indicator phenol red) was from Biochrom AG (Berlin, Germany). YNB medium or yeast nitrogen base was from Difco (Le Pont de Claix, FR), N- acetylglucosamine was from Sigma-Aldrich (St. Louis, MO, USA), and proline was from Fluka (Buchs, CH).

### 2.2. Plant Extracts and Essential Oils

Dried plants and EO of *A. indica* seeds, *C. chinensis* rhizome, *M. officinalis* bark, *S. barbata* aerial parts, and the EO of *L. scoparium* branches and twigs were acquired to Magnolien Apotheke, Germany, Karlsruher Str. 14, 69126 and kept at the Applied Physiology Laboratory (ICBAS-UP) with batch number BRR- 3576/1-5. Decoctions were prepared by adding 30 g of plant to 250 mL of distilled water, letting it soak for 45 min and simmering for 30 min. After simmering, decoctions were allowed to stand to cool and were filtered.

### 2.3. Fungal Organisms

The antifungal activity of the plant decoctions and EO was evaluated against four species of *Candida* reference strains (*C. albicans* ATCC 10231, *C. krusei* ATCC 6258, *C. parapsilosis* ATCC 22019, and *C. tropicalis* ATCC 13803) and two clinical strains (*C. glabrata* H30 isolated from a case of vaginal candidiasis and *C. albicans* H37 isolated from bronchoalveolar lavage fluid-CHSJ-Prof. Cidália Pina Vaz). *C. krusei* ATCC 6258 was also used as quality control. Germ tube inhibition assay was performed using *C. albicans* ATCC 10231. All micro-organisms were stored in Sabouraud broth medium with 20.0% glycerol at −80 °C and sub-cultured in SDA before each test to ensure optimal growth conditions and purity.

### 2.4. Susceptibility Tests

Broth microdilution methods based on the Clinical and Laboratory Standard Institute (CLSI) reference documents M27-A3 and M27-S3 with minor modifications were used to determine minimum inhibitory concentrations (MIC) [23,24]. Briefly, cell suspensions were prepared from recent cultures (24 h) of the different strains of yeasts on SDA and diluted to a final inoculum of 1–5 × 10^3^ colony forming units (CFU)/mL in RPMI-1640 broth, buffered to pH 7.0 with MOPS. The MIC of plant decoctions and EOs was determined by two-fold serial dilution. Dilutions were prepared in RPMI-1640 broth, starting from 0.3 μL/mL for each sample. Equal volume of solution samples and yeast suspensions in the test medium were then distributed into sterile 96-well plates. Sterility and growth controls in RPMI-1640 medium alone and with 2.0% of DMSO (*v*/*v*) were included. The plates were incubated in a humid atmosphere, without agitation at 35 °C for 48 h. MICs were recorded as the lowest concentrations resulting in 100.0% growth inhibition. Voriconazole MIC for *C. krusei* (ATCC 6258) was determined as quality control, and the result was within the recommended limits (data not shown) [24]. The minimum lethal concentrations (MLC) were determined after 48 h of incubation, by removing 20 µL from the MIC and highest concentration wells. The plates were incubated at 35 °C for 24 h. The MLC was defined as the lowest concentration showing 100.0% growth inhibition. All the experiments were performed in duplicates and repeated independently at least three times. 

### 2.5. Germ Tube and Dimorphic Transition Inhibition Assay

Germ tube inhibition assay was performed according to Lopes and co-workers [1] with minor modifications. *C. albicans* ATCC 10231 suspensions were obtained in YNB medium from 24 h cultures in SDA at 37 °C. The suspensions were adjusted to obtain a density of 1.0 ± 0.2 × 10^6^ CFU/mL determined by yeast counting (100 ± 20 yeasts) in the Neubauer chamber. Dilutions of the EO or decoctions were prepared and diluted in yeast suspension to obtain appropriate inhibitory and sub-inhibitory concentrations (1/2 to 1/256 of the MIC in *L. scoparium* and 2MIC, MIC, and 1/2 of the MIC in *C. chinensis*). As a positive control, a tube containing the yeast suspension and 1% of DMSO was included. After 3 h of incubation in a 37 °C water bath, without stirring, 100 cells from each sample were counted using the Neubauer chamber, and then the percentage of germ tubes was determined. The presence of germ tubes was considered positive when they had at least one length equal to or greater than the diameter of the stem cell and did not present a constriction at the point of connection to the same, characteristic of the pseudohyphae. This assay was performed three or four times for each sample.

### 2.6. Statistical Analysis

Susceptibility tests were carried out in duplicates three times and the results were presented as median values. Germ tube and dimorphic transition inhibition assay was carried out in triplicate or quadruplicate with the results presented as mean value ± standard deviation (SD). Regression analyses were performed with the Microsoft Excel 2013 software version 15.0 by Microsoft Corporation (Redmond, Washington - United States of America).

## 3. Results

### 3.1. Antifungal Susceptibility Tests

Table 1 shows the results obtained for the antifungal activity of decoctions and EO against the tested *Candida* strains. *C. chinensis* EO and decoction showed a broad-spectrum anti-*Candida* activity, including all tested species, with MIC values ranging from 0.63 to 5 µL/mL: *C. krusei* and *C. tropicalis* had the lowest values while *C. glabrata* and *C. albicans* had the highest ones. The activity was similar between EO and decoction, for all the species or strains studied. In addition, the effect observed was equal for the *C. albicans*-susceptible (ATCC) or fluconazole-resistant (H37) strains. Moreover, *C. chinensis* revealed fungicidal activity against *Candida* spp. with MLC values equal to or one log_2_ above the MICs. The exception is *C. glabrata*, showing values of MLC higher than 20 µL/mL but with changes in growth and formation of microcolonies (Table 1).

Regarding *L. scoparium* EO, activity was demonstrated against all tested pathogens, with the MIC and MLC values ranging from 10 to ≥ than 20 µL/mL: *C. glabrata* being the most susceptible species and *C. tropicalis* showing a value of MIC higher than 20 µL/mL. The MIC/MLC was lower for the *C. albicans* fluconazole-resistant strain when compared to the susceptible ATCC strain (Table 1). Decoctions of *M. officinalis, A. indica*, and *S. barbata*, as well as the respective EOs, did not show anti-*Candida* activity in concentrations equal to or less than 20 μL/mL for all the species tested (Table 1) and, for that, these extracts were disregarded for the dimorphic transition inhibition assay.

### 3.2. Germ Tube and Dimorphic Transition Inhibition Assay

Results of the inhibitory effects on the formation of *C. albicans* germ tubes when treated with the *C. chinensis* EO, its respective decoction, and *L. scoparium* EO are displayed in Table 2. *C. chinensis* EO and decoction showed some effect reducing the formation of germ tubes circa only 40.0% in the concentration corresponding to the 2MIC (10 μL/mL) in comparison to untreated control cells. The following concentrations, MIC (5.00 μL/mL) and MIC/2 (2.50 μL/mL) displayed no inhibitory effect on germ tube formation in the tested strain (Table 2).

*L. scoparium* EO displayed an important inhibitory effect on the dimorphic transition, inhibiting 100.0% at MIC/2 concentration (10.00 μL/mL) and about 90.0% up to a concentration of MIC/32 (0.63 μL/mL). From this concentration down, the percentage of germ tube formation increases gradually along the decrease of the EO concentration until MIC/256 (Table 2).

## 4. Discussion

**Figure 1:**
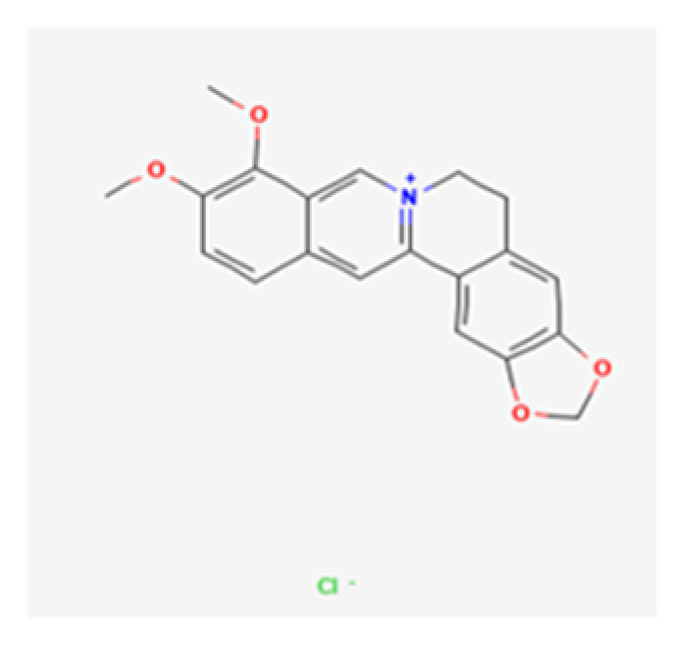
Berberine hydrochloride (PubChem: https://pubchem.ncbi.nlm.nih.gov/compund/12456, accessed on 12 September 2022).

**Figure 2:**
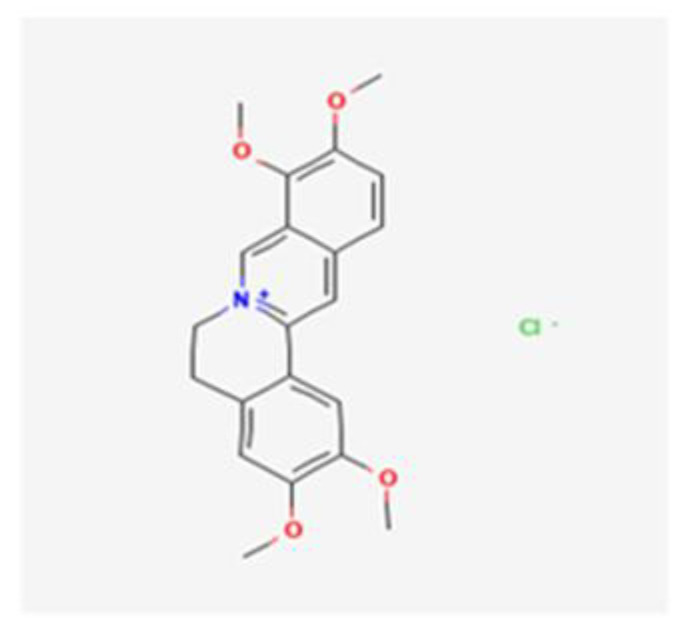
Palmatinehydrochloride (PubChem: https://pubchem.ncbi.nlm.nih.gov/compound/73442, accessed on 12 September 2022).

In TCM, herbal decoctions are specific combinations of different herbs that are used as formulas according to the patient’s symptoms and characteristics in order to treat various diseases, including infections [25]. EOs are natural products produced by aromatic plants and are rich in terpenes and terpenoids. Due to their lipophilic profile, these EOs easily integrate into the membrane structures, facilitating cellular permeability with liberation of intracellular components and possible enzymatic inactivation. EOs can act against *Candida* spp. through the inhibition of the synthesis of ergosterol and of enzymes involved in cell wall synthesis, by modifying cell wall morphology via gaining cell membrane permeability, and by producing reactive oxygen species [26,27]. *C. chinensis* is well-known for its anti-inflammatory, antioxidant, antiviral, and antimicrobial functions [25,28]. *C. chinensis* decoction and EO exhibited broad-spectrum anti-*Candida* activity: *C. albicans, C. glabrata, C. parapsilosis, C. krusei*, and *C. tropicalis* were susceptible. The results show fungicidal activity for all the species with a higher score against *C. krusei > C. tropicalis > C. parapsilosis*. The variation in susceptibility observed in the results depended on the species: *C. krusei*, which is intrinsically resistant to fluconazole [9,11], came forward as the most susceptible species, with an MIC/MLC equal to 0.63 µL/mL, and turned *C. chinensis* into a potential candidate in the treatment of candidiasis generated by this species. *C. albicans* ATCC 10231, on the other hand, was identified in the results as the least susceptible, possibly for the acquisition of resistance mechanisms, since it is the most common species and often patients are subjected to long-term treatments with antifungals [9,11]. The bioactive compounds associated with the antifungal effects could be the same in both *C. chinensis* EO and decoction. Looking closer at the similarities between their susceptibility testing results, it is possible to deduce that compounds responsible for the antifungal activity are extracted in an identical way involving temperature rising, either by the simmering in the decoction or by the steam distillation of the EO. According to previous studies, several bioactive components, including isoquinoline alkaloids such as berberine hydrochloride (Figure 1) and palmatine hydrochloride (Figure 2)—both with methoxy groups in their chemical structure—have already been identified in the *C. chinensis* rhizome [29] and proved positive antimicrobial activity [30].

**Figure 3:**
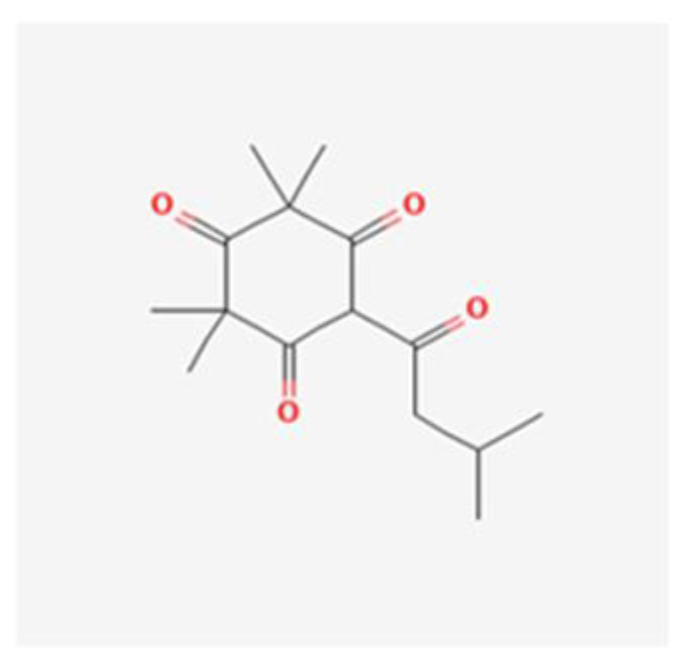
Leptospermone chemical structure (PubChem: https://pubchem.ncbi.nlm.nih.gov/compound/Leptospermone, accessed on 12 September 2022).

**Figure 4:**
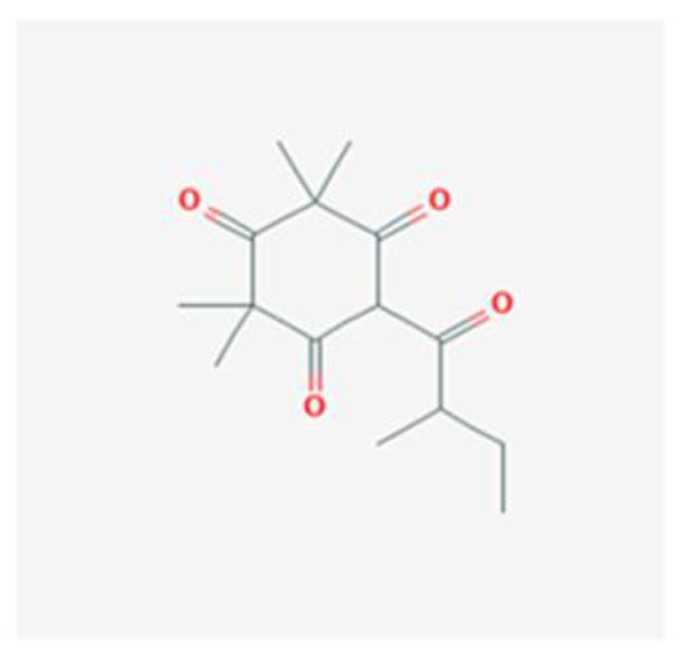
Isoleptospermone chemical structure (PubChem: https://pubchem.ncbi.nlm.nih.gov/compound/Isoleptospermone, accessed on 12 September 2022).

**Figure 5:**
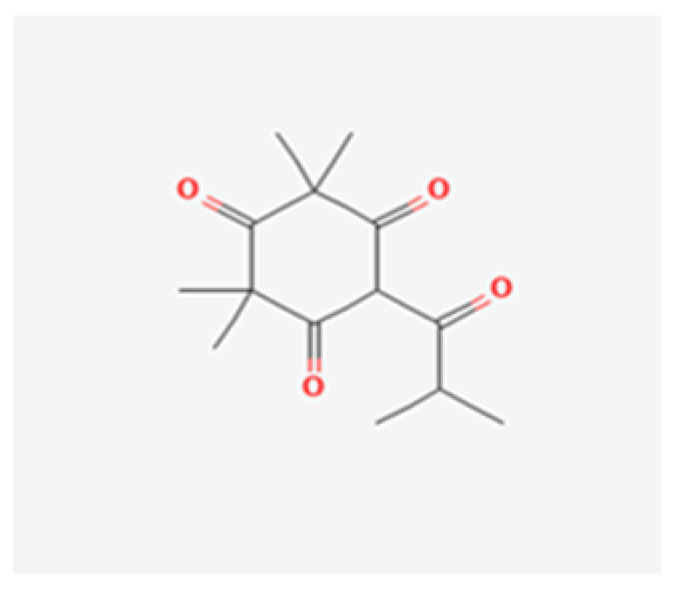
Flavesone chemical structure. (PubChem: https://pubchem.ncbi.nlm.nih.gov/compound/Flavesone, accessed on 12 September 2022).

**Figure 6:**
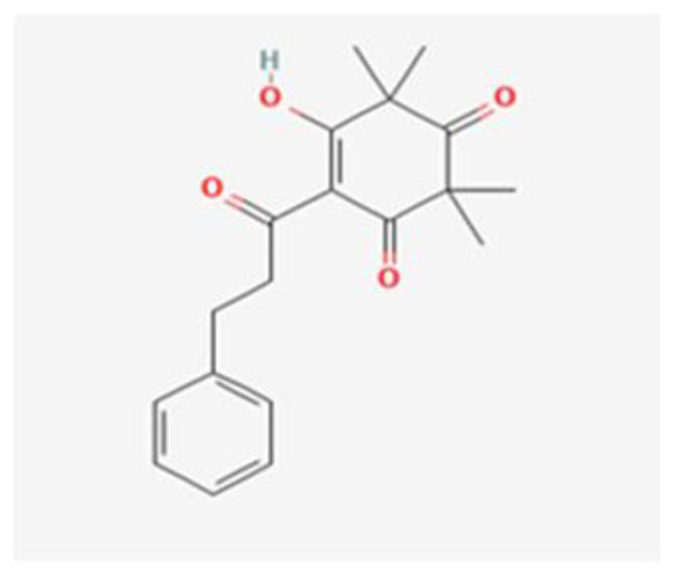
Grandiflorone chemical structure. (PubChem: https://pubchem.ncbi.nlm.nih.gov/compound/3014646, accessed on 12 September 2022).

**Figure 7:**
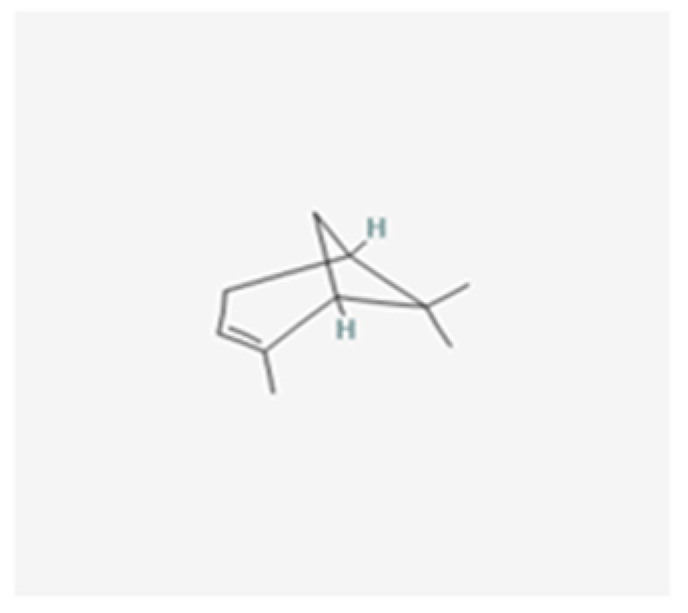
α-pinene chemical structure. (PubChem: https://pubchem.ncbi.nlm.nih.gov/compound/alpha-Pinene, accessed on 12 September 2022).

**Figure 8:**
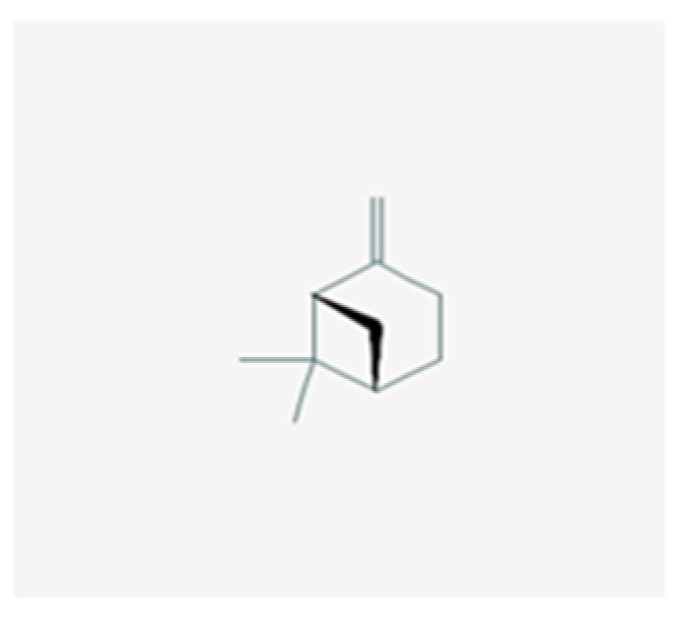
β-pinene chemical structure. (PubChem: https://pubchem.ncbi.nlm.nih.gov/compound/beta-Pinene, accessed on 12 September 2022).

Antibacterial compounds of *C. chinensis* have been isolated, identified and tested for the preservation of wood, showing favorable results with extracts in various solvents with better outcomes when isolated compounds acted in synergy [29], which can help to understand the identical results achieved by both *C. chinensis* decoction and EO in our study. Moreover, the results obtained in our study go hand in hand with those of Seneviratne et al. [8] when it comes to the antifungal action of rhizome *C. chinensis* in *C. krusei, C. glabrata*, and *C. tropicalis*, and diverge for *C. albicans* and *C. parapsilosis*, where the authors reported no antifungal activity. Additionally, the strong anti-*C.-albicans* activity demonstrated by *C. chinensis* disagrees with the study of Franzblau et al. [31]. This dissimilarity in the results translate an interest of further testing to help to understand if this difference could be justified by the fact that our study used different strains of *C. albicans* and *C. parapsilosis*. The percentage of germ tube formation by *C. albicans* was found to be reduced with both *C. chinensis* decoction and EO and a further evaluation of this result with complementary analysis could be of help to understand the mechanism of action exerted by its compounds via reduction of the adhesion properties of *C. albicans* verified in the previous literature [32], through possible downregulation of genes involved in hyphae formation, such as EFG1, HWP1, ECE1, and ALS1 [33]. *L. scoparium* EO tests indicated antifungal activity but to a lower extent than *C. chinensis*; *C. glabrata* and the fluconazole-resistant *C. albicans* strain are the most susceptible species (10 µL/mL) and *C. tropicalis* is less affected (>20 µL/mL). The action of this EO is, in general, fungistatic, with MLCs higher than the MICs. Comparing our results with several previous studies carried out with the EO of branches and twigs, the findings are contradictory. Though the activity against *C. albicans* and *C. glabrata* was similar to the results obtained in the present study [34], Chen et al. [35] reported higher MICs for *C. tropicalis* while other authors did not find activity against *C. albicans* at concentrations up to 20 µL/mL [22,36,37]. To justify the differences, we can consider the use of different *Candida* strains, the technical differences in the protocol of activity evaluation, and the different chemical composition of *L. scoparium* EO that varies according to its geographical origin, presenting several chemotypes. Although data on the anti-*Candida* activity of EOs have been summarized recently, it is often impossible to make reliable comparisons between results obtained in different studies with different methods used [27]. Moreover, often only a few EO are included in each study, adding to the fact that the botanical source, climate and environmental conditions, time of harvesting, and extraction method can affect both the composition and antimicrobial activity of commercial essential oils [27]. Altogether, these result in poor documentation of the antifungal activity of EO [27,38]. The result of the treatment may be a synergism between different effects or mechanisms of action—being known so far that triketones, mainly β-triketones, such as leptospermone (Figure 3), isoleptospermone (Figure 4), flavesone (Figure 5), and grandiflorone, (Figure 6) and monoterpenes, such as α- and β-pinene (Figure 7 and Figure 8 respectively), are the main constituents identified in *L. scoparium* EO [39]. 

Comparing the respective chemical structures between the β-triketones (Figure 3, Figure 4, Figure 5 and Figure 6) and monoterpenes (Figure 7 and Figure 8), it is possible to understand that the latter lack the element oxygen in their composition for which it may be possible that the antimicrobial action of *L. scoparium* EO is mostly due to the action of β-triketones, while the monoterpenes could be more associated to the aromatic properties. In addition to the possible activity of inhibiting yeast growth, the compounds may also act by inhibiting the virulence mechanisms, thereby decreasing the pathogenicity and reducing the progression of the infection.

Hyphal transition is considered a crucial factor in Candida infections [40] and the growth of hyphae can be induced by a pH shift or a change in temperature, with the hyphae forms being elongated and morphologically differing from round cell forms. In addition, during the growth of the hyphae, expression of new surface antigens leads to increased adhesion to host cells and facilitates tissue penetration [40]. Inhibition of morphogenesis may thus be an important target for virulence of *C. albicans* [41]. While being less active upon the growth of *C. albicans*, *L. scoparium* EO has the ability to inhibit around 90.0% the filamentation at a concentration of MIC/32 (0.63 μL/mL). Considering *C. chinensis*, there was some inhibition of germ tube formation in the concentration of 2xMIC; however, for the MIC, this inhibition ceases to occur, so the potential of this plant turns out to be more interesting due to the fungicidal activity. Within the scope of our bibliographic research, no studies performed using dimorphic transition inhibition assay in either *C. chinensis* or *L. scoparium* were found. Decoctions and EO of *M. officinalis* bark, aerial parts of *S. barbata*, and *A. indica* seeds indicate no antifungal activity for the tested concentrations. To our knowledge, there are no previous studies published considering the aforementioned parts of these plants and, for that, we lack a term of comparison for our results.

## 5. Conclusions

These results demonstrate that rhizome *C. chinensis*, EO, and decoction have relevant antifungal activity against *C. krusei > C. tropicalis > C. parapsilosis > C. glabrata > C. albicans* (fluconazole-susceptible and -resistant strains), being fungicidal in all species other than *C. glabrata*. While it does have the best results in terms of antifungal activity, *C. chinensis* does not have an important effect on germ tube formation in *C. albicans*, an important virulence factor of this species. On the other hand, EO of *L. scoparium* branches and twigs showed lower antifungal activity for *C. glabrata =* fluconazole-resistant *C. albicans* strain *> C. krusei = C. albicans* ATCC *> C. parapsilosis > C. tropicalis*. Nevertheless, this EO has a strong inhibitory effect on the germ tube formation, so further testing as a potential adjuvant with other antifungals in infections by *C. albicans*, the most frequent yeast pathogen, should be considered in future studies. The decoctions and EO of *M. officinalis* bark, aerial parts of *S. barbata*, and *A. indica* seeds showed no antifungal activity at the concentrations tested. Summing up, this work allowed us to acknowledge that, apart from the forms traditionally used in TCM, such as decoctions, EOs also have an enormous potential for use in clinical practice and increasing evidence of their antimicrobial activity. The next step of research should pass by the analysis of the antimicrobial activity of the decoctions of *C. chinensis* and *A. indica*, as well as of their EOs; *L. scoparium* EO against other fungal species rather than *Candida* spp., such as filamentous fungi; and also against both Gram-positive and Gram-negative bacteria. Furthermore, it is important to deepen the insight of the biochemical pathways implicated in the antimicrobial effect of these plants’ decoctions and EOs, as well as of their respective fractions, in order to understand which dose would be recommendable regarding safety for topical and/or oral administration. This study is a small step out of many involved in the investigation of plants of traditional use. The knowledge of traditional herbal medicine is fundamental for the (re)discovery of new therapeutic agents, making the studies in this area of research essential to justify the traditional and millenary use of many plants and to establish a solid partnership between conventional and non-conventional medicine in an innovative and complementary approach.

## Figures and Tables

**Table 1:** Antifungal activity (minimum inhibitory and minimum lethal concentrations (MIC and MLC, respectively)) of *Coptis chinensis, Magnolia officinalis, Azadirachta indica*, and *Scutellaria barbata* decoctions (D) and essential oils (EO) and *Leptospermum* *scoparium* EO against *Candida* strains.

	*C. albicans*		*C. albicans* H37 (FR)		*C. glabrata*		*C. krusei*		*C. parapsilosis*		*C. tropicalis*	
	MIC ^a^	MLC ^b^	MIC ^a^	MLC ^b^	MIC ^a^	MLC ^b^	MIC ^a^	MLC ^b^	MIC ^a^	MLC ^b^	MIC ^a^	MLC ^b^
*C. chinensis* (D)	5.00	10.00	5.00	10.00	5.00	>20.00 *	0.63	0.63	2.5	2.5	0.63	1.25
*C. chinensis* (EO)	5.00	10.00	5.00	10.00	5.00	>20.00 *	0.63	0.63	2.5	2.5	0.63	1.25
*L.scoparium* (EO)	20.00	>20.00	10.00	20.00	10.00	>20.00	20.00	≥20.00	≥20.00	>20.00	>20.00	>20.00
*M. officinalis* (D)	>20.00	-	-	-	>20.00	-	>20.00	-	>20.00	-	>20.00	-
*M. officinalis* (EO)	>20.00	-	-	-	>20.00	-	>20.00	-	>20.00	-	>20.00	-
*A. indica* (D)	>20.00	-	-	-	>20.00	-	>20.00	-	>20.00	-	>20.00	-
*A. indica* (EO)	>20.00	-	-	-	>20.00	-	>20.00	-	>20.00	-	>20.00	-
*S. barbata* (D)	>20.00	-	-	-	>20.00	-	>20.00	-	>20.00	-	>20.00	-
*S. barbata* (EO)	>20.00	-	-	-	>20.00	-	>20.00	-	>20.00	-	>20.00	-

FR—Fluconazole resistant (H37); * formation of microcolonies; ^a^ MIC was determined by microdilution method and expressed in μL/mL (*v*/*v*); ^b^ MLC was expressed in μL/mL (*v*/*v*); **-** not tested, >20 μL/mL (*v*/*v*).

**Table 2:** Percentage of germ tube formation by *Candida* *albicans* ATCC 10231 incubated with various concentrations (µL/mL) of *Coptis* *chinensis* (decoction and EO) and *Leptospermum scoparium* EO. Results presented as mean ± standard deviation of three or four experiments.

Concentration µL/mL	*C. chinensis* Decoction	*C. chinensis* EO	*L. scoparium* EO
Control	84.6 ± 8.5	84.6 ± 8.5	84.6 ± 8.5
10	64.1 ± 4.1 (2MIC)	59.3 ± 5.7 (2MIC)	0.0 ± 0.0 (MIC/2)
5	88.2 ± 3.9 (MIC)	89.4 ± 4.9 (MIC)	5.1 ± 1.2 (MIC/4)
2.5	89.4 ± 6.4 (MIC/2)	93.5 ± 3.9 (MIC/2)	8.3 ± 2.5 (MIC/8)
1.25	-	-	10.0 ± 6.2 (MIC/16)
0.63	-	-	12.0 ± 5.3 (MIC/32)
0.31	-	-	40.7 ± 8.3 (MIC/64)
0.16	-	-	76.0 ± 10.5 (MIC/128)
0.08	-	-	92.3 ± 4.9 (MIC/256)

EO–Essential oil; MIC-minimum inhibitory concentration.

## Data Availability

Not applicable.
